# Vitamin D receptor: a possible biomarker for sporadic parathyroid adenoma?

**DOI:** 10.1007/s13304-025-02182-5

**Published:** 2025-04-05

**Authors:** Angeliki Chorti, Angeliki Cheva, Kassiani Boulogeorgou, Anthoula Chatzikyriakidou, Charoula Achilla, Despoina Tsalkatidou, Despoina Krokou, Sohail Bakkar, Papavramidis Theodossis

**Affiliations:** 1 1st Propaedeutic Department of Surgery, Faculty of Health Science, Medical School, AHEPA University Hospital, Aristotle University, St.Kiriakidi 1, 54636 Thessaloniki, Greece; 2 Laboratory of Pathology, Faculty of Health Science, Medical School, Aristotle University, Thessaloniki, Greece; 3 Laboratory of Medical Biology - Genetics, Faculty of Health Science, Medical School, Aristotle University, Thessaloniki, Greece; 4 Department of General and Specialized Surgery, Faculty of Medicine, The Hashemite University, Zarqa, Jordan; 5 Minimal Invasive Endocrine Surgery Department, Kyanos Stavros, Euromedica, Thessaloniki, Greece

**Keywords:** Vitamin D receptor, Parathyroid adenoma, Parathyroid gland, Biomarker, Genetics

## Abstract

Parathyroid adenoma is the main cause of primary hyperparathyroidism. The genetic basis of the disease is still unclear. Vitamin D receptor (VDR) is involved in parathormone regulation. The aim of this study is to evaluate Vitamin D receptor expression in sporadic parathyroid adenoma. Fifty-one patients with parathyroid adenoma and 51 healthy volunteers were enrolled in the study and genetic and immunohistochemical studies were conducted. VDR polymorphism TaqI was correlated with parathyroid adenoma development, while VDR stained positive in immunohistochemical study. Our study suggests VDR as a major contributor to sporadic parathyroid adenoma formation in Greek population.

## Introduction

Primary hyperparathyroidism (PHPT) affects 1% of the population and is characterized by hypersecretion of parathormone (PTH) and hypercalcemia. The main cause of PHPT is parathyroid adenoma, which is in the majority of cases sporadic (90%) [1, 2]. The genetic basis of parathyroid adenoma development is yet obscure. Cell regulatory, apoptotic angiogenic and growth factor genes have been correlated to the development of parathyroid adenoma [3]. Furthermore, an extensive number of proteins have altered expression patterns in parathyroid adenomas [4–8].

Vitamin D Receptor (VDR) acts as calcitriol mediator to the nucleus, regulating PTH production [9]. VDR is an intracellular receptor that acts as a nuclear transcription factor which forms a complex with 1,25(OH)2D3, hormonally active form of vitamin D, and regulates activation or repression of many target genes. VDR is active in a wide variety of tissues including colon, breast, lung, ovary, bone, kidney, parathyroid gland, pancreatic b-cells, monocytes, T lymphocytes, melanocytes, keratinocytes [10]. According to the existing literature, VDR immunohistochemical staining is nuclear in classic Hodgkin lymphoma cells, although cytoplasmatic reaction was also tested [11]. Moreover, nuclear and cytoplasmatic VDR staining was observed in endometrioid cancer cells, whereas in urinary bladder carcinoma, VDR staining was cytoplasmatic and membranous [12, 13].

The authors believe in the clonic nature of the basis of parathyroid adenomas.

In this context, we targeted sign molecule pathways for adenomas. Specifically, the aim of our pilot study is to evaluate Vitamin D receptor gene expression pattern in parathyroid adenoma patients in Greek population.

## Methods

This study included two groups of participants: (a) patients’ group and (b) control group. Patients that underwent surgery for sporadic parathyroid adenoma from 2019 to 2022 in our Surgical Department were enrolled in this study (Group patients). The control group conducted by healthy volunteers with no personal or family history of chronic autoimmune or neoplastic diseases, so that there is no interference with other dysregulated genes. The inclusion criteria included: (a) participants > 18 years old, (b) patients with sporadic parathyroid adenoma underwent parathyroidectomy, (c) no personal or family history of chronic autoimmune or neoplastic diseases. Exclusion criteria were: (a) patients with secondary or tertiary hyperparathyroidism and (b) patients with parathyroid hyperplasia or carcinoma. Participants in both groups were matched for demographic characteristics (age, gender). An ethics approval was obtained for this study by the Local Ethical Committee and informed written consent was obtained by each participant.

Our study included two different parts: the first part included the genetic study of VDR gene polymorphism and the second part referred to the immunohistochemical study of VDR in parathyroid adenoma samples. These two parts were conducted distinctly and the two different scientific groups were blinded to the other part results.

Blood samples were obtained from both study groups and genomic DNA was extracted from peripheral blood lymphocytes using the PureLink Genomic DNA Kit (Invitrogen, Karlsruhe, Germany). VDR gene polymorphisms TaqI, ΒsmI, FokI and ApaI were studied using polymerase chain reaction–restriction fragment length polymorphism (PCR–RFLP) assay, as described previously [14].

Regarding second part, only patients’ group participants were included. Parathyroid tissue samples from Formalin-Fixed Paraffin Embedded blocks were extracted and stained with Hematoxylin–Eosin (H&E) as well as sectioned on positive charged slides for immunohistochemical stains. VDR, rabbit polyclonal antibody (SANTA CRUZ USA) at a 1:50 dilution was applied and the tissue staining was assessed for each distinct parathyroid cell type. The intensity of staining (negative, soft, moderate, intense) and the type of staining (nuclear, cytoplasmic, membranous) were assessed. A percentage > 5% was considered as positive result.

### Statistical analysis

Quantitative variables that follow a normal distribution were reported as means ± standard deviation or median (interquartile range) for those that do not follow a normal distribution. Qualitative variables were reported as frequencies and relative frequencies. Sample normality was tested using the Kolmogorov–Smirnov test. Pearson’s chi-square test was used to test differences in polymorphism distribution between PHPT patients and controls. When expected values were less than 5, Fisher’s exact test was used instead. The correlation between categorical and continuous variables was tested with Kruskal–Wallis test. A difference at *p* ≤ 0.05 was considered as statistically significant in all statistical tests. All the analyses were performed using SPSS statistical package 24.0 (IBM Corp., Armonk, NY, USA).

## Results

The clinicopathological characteristics of patients’ and control group are presented in Table 1. The male-to-female ratio was 1:50 for patients and 4:47 for control group, while mean age had no statistically significant difference between groups (*p* = 0.96).

**Table 1:** Clinicopathological characteristics of study participants

	Patient group	Control group	*p*-value
Age (years old)	54.11 ± 12.46	50.43 ± 9.36	0.96
Gender (Male:Female)	1:50	4:47	
Preoperative serum calcium (mg/dl)	10.91 ± 0.76	n/a	
Preoperative serum parathyroid hormone (pg/ml)	82.77 ± 21.04	n/a	
Preoperative serum phosphorus (mg/dl)	2.88 ± 0.46	n/a	
Postoperative serum calcium (mg/dl)	9.12 ± 0.62	n/a	
Postoperative serum parathyroid hormone (pg/ml)	18.11 ± 16.99	n/a	
Postoperative serum phosphorus (mg/dl)	3.38 ± 0.74	n/a	
Adenoma maximal dimension (cm)	1.87 ± 0.73	n/a	
Adenoma weight (gr)	1.12 ± 1.04	n/a	

*n/a* not applicable

Regarding genetic study, only VDR gene polymorphism TaqI TC and TT genotypes were correlated to sporadic parathyroid adenoma development concerning additive and recessive models (Table 2). When the studied polymorphisms were analyzed in pairs for their association with PHPT risk, statistical significance in genotype distributions between patients and controls remained for the ΒsmI-TaqI, FokI-TaqI, and ApaI-TaqI pairs (Table 3).

**Table 2:** Statistical analysis of VDR gene/TaqI polymorphism between patients with primary hyperparathyroidism and controls

TaqI (rs731236)		
TT	Additive (TT *vs*. Tt *vs*. tt)	**0.02**
Tt + TT	Recessive (tt *vs*. Tt + TT)	**0.04**

Bold: *p* values are statistically significant

**Table 3:** Genotype distributions between patients and controls for the FokI-TaqI, ApaI-TaqI and ΒsmI-TaqI polymorphisms’ pairs

Polymorphism Pair	*p*-value
FokI_TaqI	**0.04**
ApaI_TaqI	**0.03**
BsmI_TaqI	**0.04**

Bold: *p* values are statistically significant

**Fig. 1:**
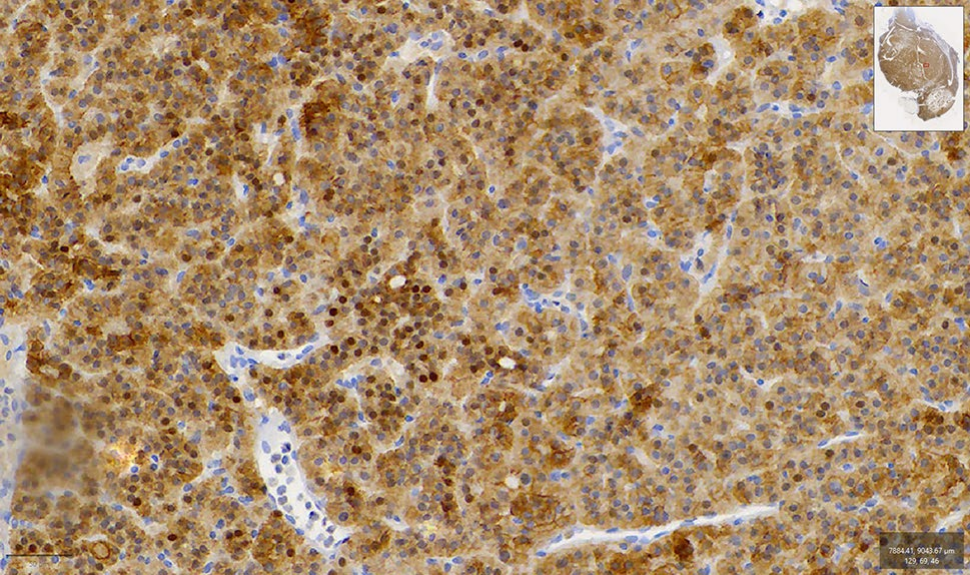
Positive cytoplasmic, nuclear and membranous types of immunohistochemical staining for VDR gene

**Fig. 2:**
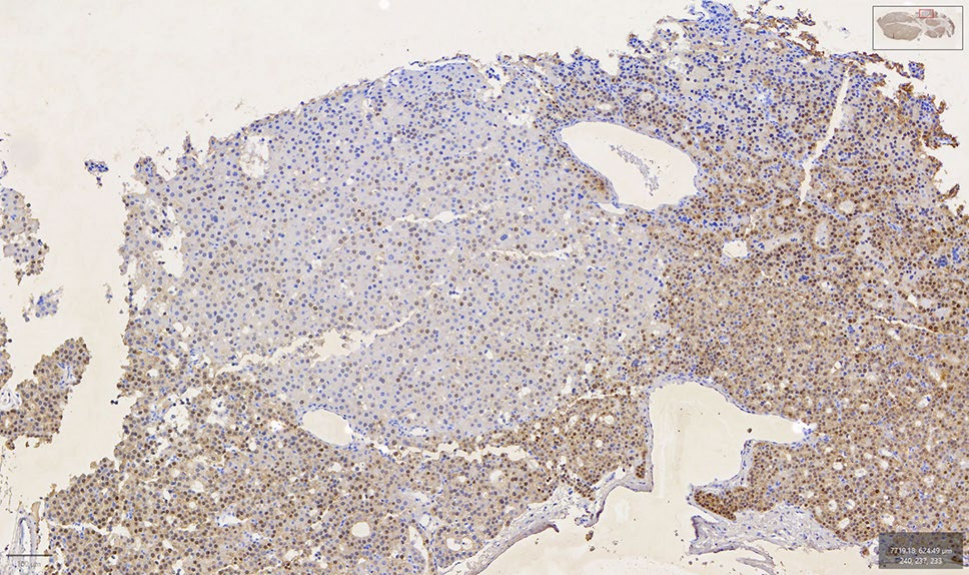
Nodule-forming cells with different immunohistochemical staining compared to the surrounding cells’ immunohistochemical pattern

The immunohistochemical staining for VDR was positive in 22.8% of the examined tissue samples regarding nuclear staining. When the overall rate of positive staining was evaluated, including cytoplasmic, nuclear and membranous types, it was found 78.5%, proposing a potential novel immunohistochemical pattern for VDR expression in parathyroid adenoma cells (Fig. 1). A clearly distinct immunophenotype was observed in the same adenoma, with groups of nodule-forming cells being either negative in staining or of mild intensity compared to the rest of the adenoma that had an intense reaction (Fig. 2).

## Discussion

Vitamin D Receptor was found to be linked to sporadic parathyroid adenoma development by distinct genetic and immunohistochemical studies. VDR expression has been found downregulated in parathyroid adenomas [15].

The results of our genetic study demonstrated VDR polymorphism TaqI as a cofounding factor for parathyroid adenoma formation [16]. In previous studies, VDR polymorphisms have been found to affect positively parathyroid hormone regulation in different populations such as Caucasian women and Iranian hemodialysis patients [17, 18].

The immunohistochemical staining of parathyroid adenoma was found positive in variable intensity and in different types (nuclear, membranous, cytoplasmatic) [19]. Two other studies demonstrated mild-to-moderate intensity of VDR staining in adenomas compared to normal tissue samples [5, 20].

What is of great importance, is the distinct immunophenotype observed in certain cases with the nodule-forming cells appearing with different intensity from the surrounding cells. This implies a novel pattern of sporadic parathyroid adenoma formation with similar certain pathways, suggesting a common genetic developmental model among sporadic cases.

Our study demonstrates certain limitations, as the small sample (only 51 Greek patients), which has certainly impact on the results exported, and larger studies are mandatory to validate these findings. We conducted a pilot study and further analysis will be held.

Concluding, this study confirms the contribution of vitamin D receptor in sporadic parathyroid adenoma development in Greek population.

## References

[1] Erickson LA, Mete O, Juhlin CC et al (2022) Overview of the 2022 WHO classification of parathyroid tumors. Endocr Pathol 33:64–89. 10.1007/s12022-022-09709-1 35175514 10.1007/s12022-022-09709-1

[2] Cetani F, Pardi E, Borsari S, Marcocci C (2011) Molecular pathogenesis of primary hyperparathyroidism. J Endocrinol Invest 34:35–39 21985978

[3] Mizamtsidi M, Nastos C, Mastorakos G et al (2018) Diagnosis, management, histology and genetics of sporadic primary hyperparathyroidism: old knowledge with new tricks. Endocr Connect 7:R56–R68 29330338 10.1530/EC-17-0283 PMC5801557

[4] Lu M, Kjellin H, Fotouhi O et al (2018) Molecular profiles of oxyphilic and chief cell parathyroid adenoma. Mol Cell Endocrinol 470:84–95. 10.1016/j.mce.2017.10.001 28986304 10.1016/j.mce.2017.10.001

[5] Akpinar G, Kasap M, Canturk NZ et al (2017) Proteomics analysis of tissue samples reveals changes in mitochondrial protein levels in parathyroid hyperplasia over adenoma. Cancer Genom Proteom 14:197–211. 10.21873/cgp.20031 10.21873/cgp.20031 PMC5420820 28446534

[6] Arya AK, Bhadada SK, Singh P et al (2019) Quantitative proteomics analysis of sporadic parathyroid adenoma tissue samples. J Endocrinol Invest 42:577–590. 10.1007/s40618-018-0958-1 30284223 10.1007/s40618-018-0958-1

[7] Donadio E, Giusti L, Cetani F et al (2011) Evaluation of formalin-fixed paraffin-embedded tissues in the proteomic analysis of parathyroid glands. Proteome Sci. 10.1186/1477-5956-9-29 21651755 10.1186/1477-5956-9-29 PMC3123619

[8] Giusti L, Cetani F, Ciregia F et al (2011) A proteomic approach to study parathyroid glands. Mol Biosyst 7:687–699. 10.1039/c0mb00191k 21180715 10.1039/c0mb00191k

[9] Demay MB, Kiernan MS, DeLuca HF, Kronenberg HM (1992) Sequences in the human parathyroid hormone gene that bind the 1,25-dihydroxyvitamin D3 receptor and mediate transcriptional repression in response to 1,25-dihydroxyvitamin D3. Proc Natl Acad Sci 89:8097–8101. 10.1073/pnas.89.17.8097 1325645 10.1073/pnas.89.17.8097 PMC49863

[10] Gnagnarella P, Raimondi S, Aristarco V et al (2020) Vitamin D receptor polymorphisms and cancer. Adv Exp Med Biol 268:53–114 10.1007/978-3-030-46227-7_4 32918214

[11] Gupta GK, Gupta GK, Agrawal T et al (2020) Immunohistochemical expression of vitamin D receptor and forkhead box P3 in classic hodgkin lymphoma: correlation with clinical and pathologic findings. BMC Cancer. 10.1186/s12885-020-07026-6 32513132 10.1186/s12885-020-07026-6 PMC7282135

[12] An HJ, Song DH (2019) Displacement of vitamin D receptor is related to lower histological grade of endometrioid carcinoma. Anticancer Res 39:4143–4147. 10.21873/anticanres.13573 31366499 10.21873/anticanres.13573

[13] Rehab Mohamed Sharaf E, Basma Mostafa Mahmoud A, Samia Ibrahim EN, Wesam Ismail M (2022) Expression of vitamin D receptor (VDR) in urinary bladder carcinoma: immunohistochemical and histopathological study. Int J Pathol Clin Res. 10.23937/2469-5807/1510139

[14] Zacharioudaki M, Messaritakis I, Galanakis E (2021) Vitamin D receptor, vitamin D binding protein and CYP27B1 single nucleotide polymorphisms and susceptibility to viral infections in infants. Sci Rep 11:13835. 10.1038/s41598-021-93243-3 34226633 10.1038/s41598-021-93243-3 PMC8257681

[15] Carling T, Rastad J, Åkerström G, Westin G (1998) Vitamin D receptor (VDR) and parathyroid hormone messenger ribonucleic acid levels correspond to polymorphic VDR alleles in human parathyroid tumors^1^. J Clin Endocrinol Metab 83:2255–2259. 10.1210/jcem.83.7.4862 9661591 10.1210/jcem.83.7.4862

[16] Chorti A, Achilla C, Tsalkatidou D et al (2023) A pilot study of the association VDR polymorphisms with primary hyperparathyroidism. In Vivo (Brooklyn) 37:1111–1116. 10.21873/invivo.13186 10.21873/invivo.13186 PMC10188045 37103067

[17] Pourfarzam M, Nia K, Atapour A, Sadeghi HM (2014) The influence of BsmI and TaqI vitamin D receptor gene polymorphisms on the intensity of hyperparathyroidism in Iranian hemodialysis patients. Adv Biomed Res 3:213. 10.4103/2277-9175.143260 25371870 10.4103/2277-9175.143260 PMC4219211

[18] Carling T, Kindmark A, Hellman P et al (1997) Vitamin D receptor allelesb, a, andT: risk factors for sporadic primary hyperparathyroidism (HPT) but not HPT of uremia or MEN 1. Biochem Biophys Res Commun 231:329–332. 10.1006/bbrc.1997.6086 9070272 10.1006/bbrc.1997.6086

[19] Cheva A, Chorti A, Boulogeorgou K et al (2024) Sporadic parathyroid adenoma: a pilot study of novel biomarkers in females. Medicina (Lithuania). 10.3390/medicina60071100 10.3390/medicina60071100 PMC11279064 39064529

[20] Rao S, Han PER et al (2000) Reduced vitamin D receptor expression in parathyroid adenomas: implications for pathogenesis. Clin Endocrinol (Oxf) 53:373–381. 10.1046/j.1365-2265.2000.01081.x 10971456 10.1046/j.1365-2265.2000.01081.x

